# Neurons with larval synaptic targets pioneer the later nervous system in the annelid *Malacoceros fuliginosus*

**DOI:** 10.3389/fnins.2024.1439897

**Published:** 2025-01-13

**Authors:** Anna Seybold, Suman Kumar, Sharat Chandra Tumu, Harald Hausen

**Affiliations:** ^1^Michael Sars Centre, University of Bergen, Bergen, Norway; ^2^Institute of Zoology, University of Innsbruck, Innsbruck, Austria; ^3^Department of Biosciences, University of Oslo, Oslo, Norway; ^4^Department of Earth Sciences, University of Bergen, Bergen, Norway

**Keywords:** nervous system, development, evolution, neural circuitry, volume electron microscopy, Annelida, lophotrochozoa, larvae

## Abstract

Comparative studies on the development of nervous systems have a significant impact on understanding animal nervous system evolution. Nevertheless, an important question is to what degree neuronal structures, which play an important role in early stages, become part of the adult nervous system or are relevant for its formation. This is likely in many direct developers, but it is not the case in forms with catastrophic metamorphosis. It is not clear in many forms with gradual metamorphosis. This introduces uncertainty in tracing the evolution of nervous systems and of larval forms. One of the prominent larval characteristics of numerous planktonic marine organisms is the epidermal ciliation used for swimming and steering, which disappears during metamorphosis. Therefore, the neuronal elements controlling the ciliary beating are often assumed to vanish with the cilia and regarded as purely larval specializations. With volume EM, we followed the neuronal targets of the very first pioneer neurons at the apical and posterior ends of the larva of the annelid *Malacoceros fuliginosus*. We observed that all of these pioneers appear to have a dual function. Although they are laying the paths for the later adult nervous system, they also make synaptic contact with the main ciliated ring of the larva. We propose that the formation of the later adult nervous system and the innervation of the larval locomotory organ are indeed closely linked to each other. This has implications for understanding the early nervous system development of marine larvae and for existing hypotheses on nervous system evolution.

## Introduction

Nervous system development is a multifaceted process that includes the specification, patterning, and differentiation of individual neurons, the targeted outgrowth of neurites, and the formation of neuronal contacts and circuits. In animals that have a centralized nervous system or at least bundles of neurites, pioneer neurons play an important role, as they are the first to send out axons over long distances (Bate, 1976; Imai and Sakano, 2011; Raper and Mason, 2010). These neurites form a framework that is used for orientation by the growing neurites of many of the neurons, forming later the follower neurons.

The outgrowth of neurites starts early during development, as soon as the first neurons are specified and start differentiating. In direct developers, the main components of the later adult nervous system are formed immediately, whereas in indirect developers, certain parts of the nervous system may only serve functions in the larval phase and may not survive metamorphosis (Hartenstein, 2018; Schmidt-Rhaesa et al., 2015; Wanninger, 2008). This is most evident in organisms that undergo drastic changes during metamorphosis, losing large parts of the larval nervous system, as seen in phoronids (Santagata, 2002; Temereva and Wanninger, 2012) or forming most parts of the adult nervous system anew, as observed in insects (Lee and Park, 2021; Yaniv and Schuldiner, 2016), echinoderms (Formery et al., 2021; Hinman and Burke, 2018), or recently branched nemerteans (Maslakova, 2010). In contrast, in many larvae from groups such as mollusks (Croll, 2009), annelids (Fischer et al., 2010; Meyer et al., 2015), or early branched nemerteans (Von Döhren, 2024), the body organization and the nervous system during metamorphosis change more gradually.

For these forms with gradual metamorphosis, it is often unclear which neurons have a purely larval-specific function and do not become an integral part of the adult nervous system. Moreover, it is known to what extent the development of larval-specific and adult elements of the nervous system depends on each other. Closer insights into these topics would improve the understanding of larva- and adult-specific traits in neural development, neurobiology, and behavior. It would also be interested in the context of ongoing debates on how animal life cycles and body organization evolved, such as whether the adult body organization derived from a larva-like organism (Marlow et al., 2014; Martín-Zamora et al., 2023; Nielsen, 2013; Peterson et al., 1997) or vice versa (Page, 2009; Raff, 2008; Wang et al., 2020).

For getting closer insights into the relationship between the larval and adult nervous systems, studies on the neural elements of the larval epidermal ciliation are well suited. The epidermal ciliary bands used for swimming are one of the most obvious larval features in many marine animals (Nielsen, 2004, 2005). The bands are underlined by neurites, which can modulate their beating pattern and mediate behavioral reactions to environmental stimuli in stages where musculature is not yet or only partly involved (Alfaro et al., 2014; Bezares Calderón et al., 2023; Bezares-Calderón et al., 2018; Braubach et al., 2006; Jékely et al., 2008; Penniman et al., 2013; Thiel et al., 2019; Verasztó et al., 2017). During metamorphosis, the animals lose most, if not all, epidermal ciliary bands, and the associated neurites also disappear.

Whether this implies that, in organisms with gradual metamorphosis, the early neurons with extending neurites die, or whether these cells persist and maintain contacts with other targets that are lasting, has not yet been systematically studied. Synaptic connectivity data from 3-day-old annelid *Platynereis dumerilii*, however, render a set of 8 ciliomotor cells as likely purely larval neurons. These cells (MC, cMN, Ser-h1, MNl3, and MNr3) exclusively target and regulate the beating of the prototroch ciliary band cells (Jékely et al., 2024; Verasztó et al., 2017), which is ultimately lost a few days later during metamorphosis. In contrast, other ciliary bands, which survive metamorphosis, such as the paratrochs in the trunk region (Fischer et al., 2010), are targeted by a distinct set of ciliomotor cells (Jékely et al., 2024; Verasztó et al., 2017). Therefore, the larval set of neurons controlling the prototroch can be regarded as a unit, which is largely independent of the neurons that constitute the later nervous system. It is, however, unknown where and when prototroch-specific ciliomotor neurons arise and how their neurites find their way to the prototroch.

Therefore, in this study, we investigated the onset of nervous system development in an annelid, focusing on which neurons make initial contact with the prototroch and how these cells relate to the adult nervous system. We selected the annelid *Malacoceros fuliginosus* as the study subject for two reasons. 1) On the light microscopic level, the first neurons sending long neurites across the body have been identified and characterized in this species (Kumar et al., 2020). 2) The phylogenetic positions of *M. fuliginosus* and *P. dumerilii* in the two largest groups of annelids (Struck et al., 2011; Weigert and Bleidorn, 2016) add a comparative and evolutionary aspect.

The expression of proneural genes in *M. fuliginosus* begins as early as 5 h post fertilization (hpf), and a few hours later, the first neuronal cells start appearing at the anterior, posterior, and along the prototroch (Kumar et al., 2020). A posterior peripheral sensory neuron (PPN) sends two axons anteriorly, which pioneer the posterior ventral nerve cord. An anterior ganglion cell (AN1) sends an axon down on the right body side toward the developing ventral nerve cord, and two ventral bi-axonal cells prefigure the course of the nerve underlying the prototroch. Around 20 hpf, the axons of the PPN and AN1 reach the level of the prototroch. With volume EM, we investigated the synaptic targets of these cells at 22 and 28 hpf with the intention of finding the neurons suggested to be pioneer neurons in Kumar et al. (2020) and to investigate whether they made any connection to the ciliary band cell. Two phases were analyzed to monitor potential differences and to complement each other. They were chosen based on a previous study by Kumar et al. (2020), where pioneer neurites appear close to their target from 20 hpf on. Following this study, we observe that the formation of the later adult nervous system and the innervation of the larval locomotory organ are indeed closely related. We discuss the implications for early larval behavior and the evolution of both nervous systems and larval forms.

## Materials and methods

### Animal material

Individual females and males of adult *M. fuliginosus* were collected from a culture that had been maintained in the laboratory for several years. After several rinses with filtered seawater, the animals remained in separate bowls until they spawned. Staging begins from the time the gametes were combined in a fresh bowl. The embryos developed at 18°C under a light–dark 12:12 h cycle and with water changes every day or every second day. The larvae were fed with the microalgae *Chaetoceros calcitrans* from 24 hpf onward after each water change.

### *In situ* hybridization

The larvae were collected with nets and rinsed with fresh seawater. Fixation and *in situ* hybridization were performed according to the protocol described in Kumar et al. (2020). All probes were DIG-labeled and detected by Fast Blue (Sigma) staining according to the protocol described in Lauter et al. (2011). Specimens were stained for up to 24 h before preparing for imaging as described below.

### Immunolabeling

The immunolabeling was performed according to the protocol described in Kumar et al. (2020). Briefly, 22- and 28-h-old larvae were first relaxed in 1:1 MgCl_2_-seawater for 3–5 min. All animals were fixed in 4% PFA (in 1X PBS, 0.1% Tween20) for 30 min. After fixation, samples were washed twice in PTW and twice in THT (0.1 M Tris pH 8.5, 0.1% Tween20). Blocking was in 5% sheep serum in THT for 1 h before incubating in primary antibodies (monoclonal anti-acetylated *α*-tubulin, 1:300 Sigma product number T6793) for 48 h at 4°C. The samples were washed 2x for 10 min in 1 M NaCl in THT and 5x for 30 min in THT before incubating in secondary antibodies (Alexa Fluor 1:500, Thermo Fisher Scientific) at 4°C overnight. Next, the samples were washed in THT, 2x for 5 min and 5x for 30 min. Specimens were stored in embedding medium (90% glycerol, 1x PBS, and 2% DABCO) at 4°C.

### Light microscopy and image processing

The samples were mounted in glycerol and imaged using a Zeiss Examiner A1 microscope. Confocal imaging was performed using a Leica SP5 microscope. Images were processed using ImageJ, Adobe Photoshop CS6, and Imaris (Bitplane) and subsequently assembled using Adobe Illustrator CS6.

### Electron microscopy and image processing

For chemical fixation, 22- and 28-hpf larvae were relaxed for 5 min in 7% MgCl_2_ and seawater mixed 1:1 and then fixed in 2.5% glutaraldehyde in 0.1 M PBS + 9% sucrose (to achieve mOsm~1,050, similar to seawater). They were postfixed with 1% osmium tetroxide in the same buffer, dehydrated with a graded acetone series, and embedded in Epon/Araldite. All steps were performed in a microwave oven (PelcoBioWave^®^Pro +, Ted Pella, Redding, CA, USA). Serial sections of 50 nm were cut using an ultra 35° diamond knife (Diatome, Biel, Switzerland) on a UC7 ultramicrotome (Leica) and then collected on Beryllium-Copper slot grids (Synaptek, Reston, VA, USA) coated with 1% polyetherimide (in chloroform) and contrasted with 2% uranyl acetate (in water) and lead citrate. A complete series comprising 613 slices (22 hpf) and 619 slices (28 hpf) with an average FoV of 200×100 μm was imaged using STEM-in-SEM as described by Kuwajima et al. (2013) at a resolution of 4 nm/pixel using a Supra 55VP (Zeiss, Oberkochen, Germany) equipped with Atlas (Zeiss) for automated large field of view imaging. Acquired images were processed with Adobe Photoshop CC, chopped to 4096 × 4096 via Matlab for further faster processing, and then registered rigidly, followed by affine and elastic alignment with TrakEM2 (Cardona et al., 2012) implemented in Fiji (RRID:SCR_002285). The sectioning was performed longitudinally from dorsal to ventral, through the entire thickness of the larvae, with a total section loss of approximately 3% in both stages.

## Results

### General morphology of the larval central nervous system at EM level

**Figure 1:**
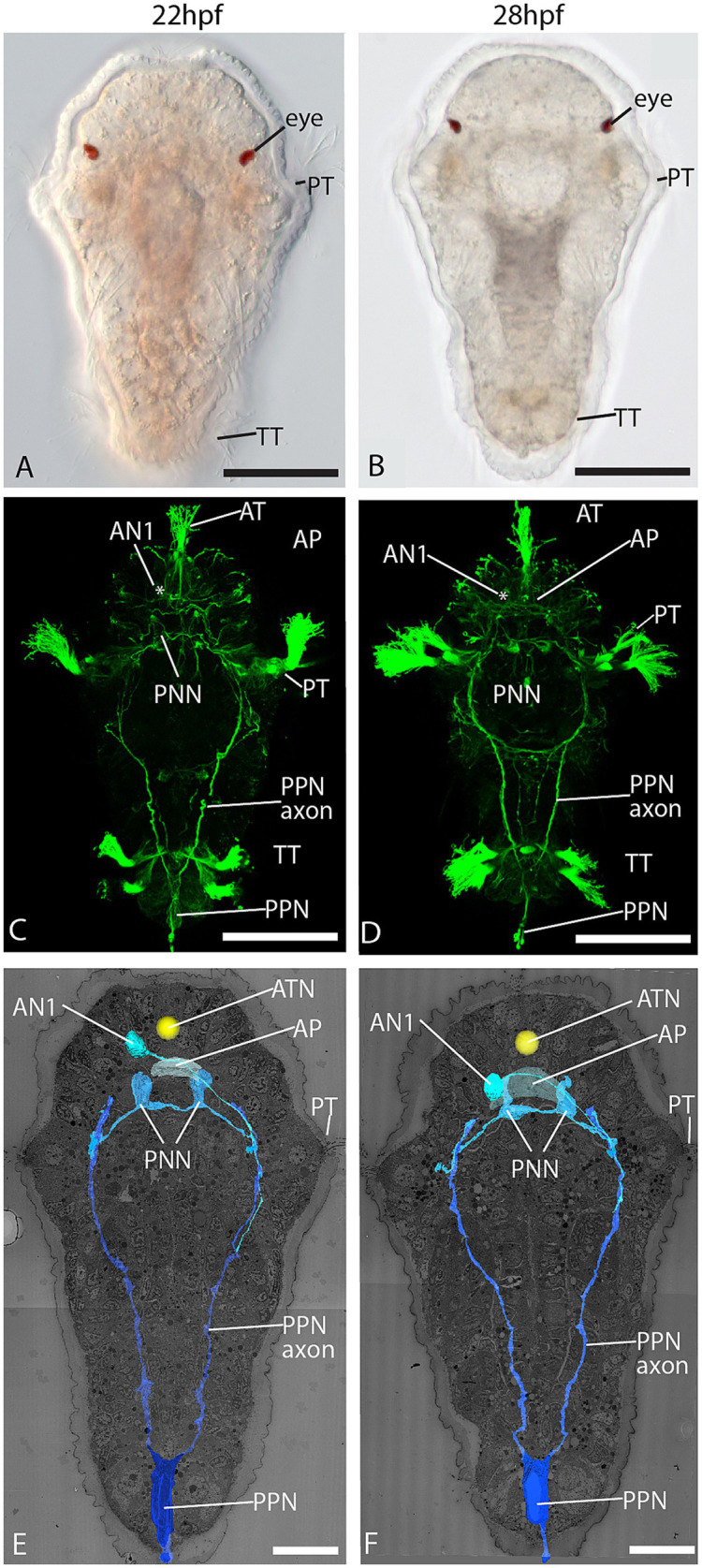
Dorsal view of 22 and 28 hpf larval stages. **(A,B)** LM; **(C,D)** confocal laser scanning microscopy (CLSM) of acetylated α-tubulin immunolabeling; and **(E,F)** 3D reconstruction of an electron microscopic dataset showing the pioneer neurons PPN, PNN, and AN1. AN1 = anterior neuron 1, AP = apical plexus, AT = apical tuft, ATN = apical tuft nucleus, PNN = prototroch nerve forming neuron, PPN = posterior pioneer neuron, PT = prototroch, TT = telotroch, arrows = epidermal sensory cells. Scale bar: **(A-D)**, 50 μm; **(E,F)** 20 μm.

By studying whole-body EM datasets of 22 and 28 h post-fertilization (hpf) larvae of *Malacoceros fuliginosus*, we could identify all major elements of the early nervous system described with light microscopy by Kumar et al. (2020) and other typical landmarks of annelid trochophore larvae on the ultrastructural level. The ciliary bundle of the larval apical tuft is formed by one large multiciliated cell with an extraordinarily large nucleus and strong basal pigmentation (Figures 1A–F; Supplements 1A,B). Underneath this cell lies the apical plexus, the crisscrossing neurites of the prospective larval brain (Figures 1C–F; Supplements 1B,C). The plexus grows considerably from 22 hpf to 28 hpf in size (~3.6 μm versus ~6.5 μm in anteroposterior extension) and number of neurites. Basal processes emerge from the apical tuft cell and cross the apical plexus. While we could observe synaptic input to the tuft cell processes (Supplement 1D), we could not observe any presynaptic terminals within the cell. The primary ciliary band, namely the anterior prototroch, is well-developed in both stages (Figures 1A–F). Multiciliated cells form a transversal semicircle, which is closed on the ventral side and open on the dorsal side. The telotroch on the posterior end is also present and composed of a few multiciliated cells. With both electron and light microscopy, we identified numerous sensory cells scattered across the epidermis in front of the prototroch and sending short neurites toward the apical plexus (Figures 1C,D; Supplements 2A–C) as it has been observed by Kumar et al. (2020).

Yet, we observed only a few cells sending out long neurites that travelled across different body regions. These cells correspond in position and course of neurites to the early pioneer neurons described by Kumar et al. (2020), which outline the major routes of the central nervous system. One neuron at the posterior end sends out two long axons running anteriorly. Based on the corresponding position and the course of the neurites, we identified this neuron as the posterior pioneer neuron (PPN), which prefigures the course of the paired posterior ventral nerve cord (Figures 1C–F). No other neuron with these characteristics was observed in this region, either with EM or LM. The few other neurites we observed running along the axon of the PPN are thought to be axons of follower neurons populating the ventral nerve cord. Two sensory cells at the ventral side of the prototroch send out neurites underlying the prototroch cells and were identified as the prototroch nerve-forming neurons (PNNs) described by Kumar et al. (2020) (Figures 1C–F). One cell with an axon connecting the brain region with the trunk nervous system could be identified as the anterior neuron 1 (AN1).

### The prototroch cells send long intermingling processes toward neural structures

**Figure 2:**
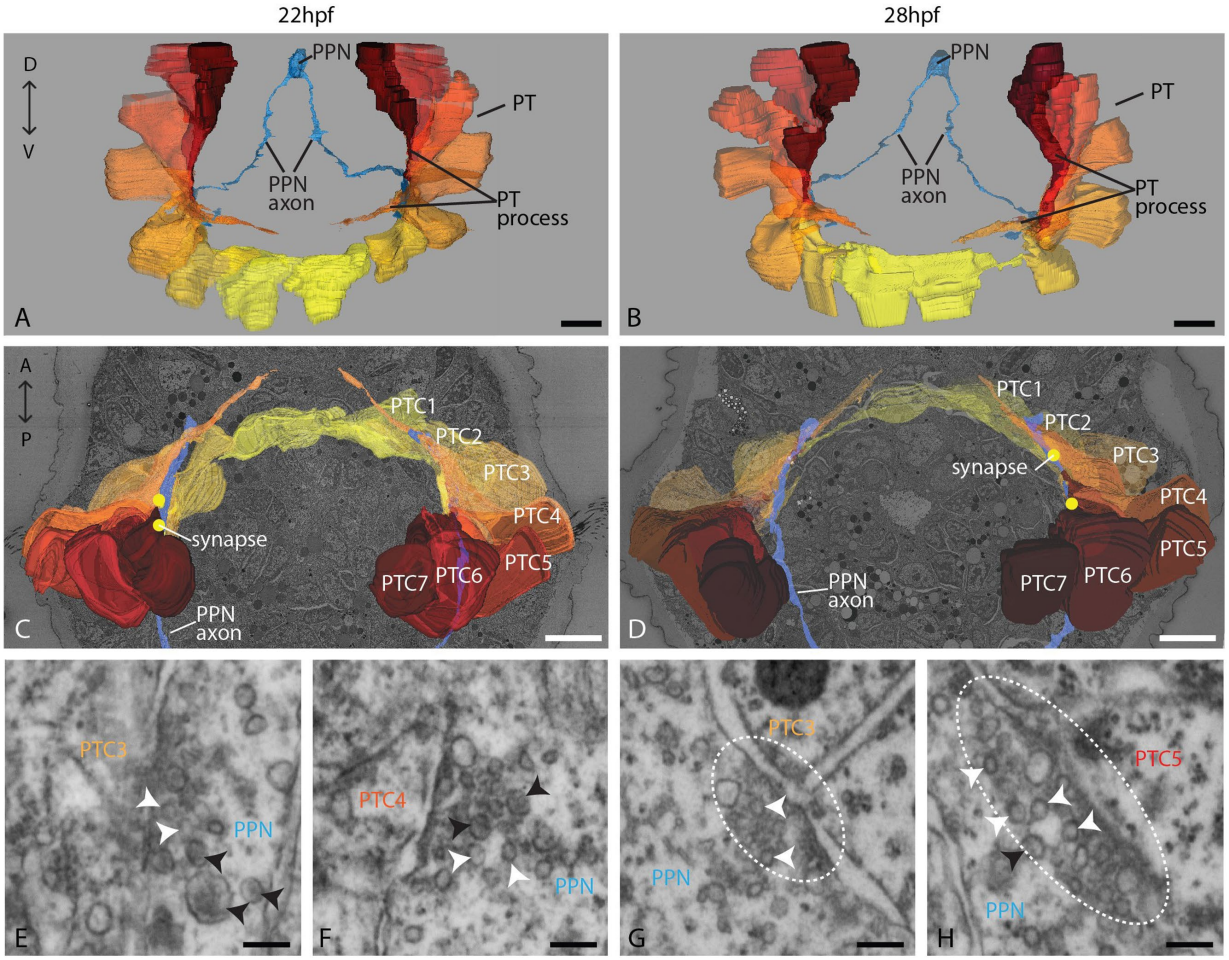
Organization of the PPN and the prototroch at 22 and 28 hpf. **(A,B)** An anterior overview of 3D reconstructions of the PPN (blue) and the prototroch cells 1–7 (yellow to red); **(C,D)** a dorsal view of 3D reconstructions of PPN, prototroch cells, and selected synapses (yellow dots); and **(E–H)** detail of selected synapses containing clear and dense core vesicles (white and black arrowheads, respectively) between PPN and prototroch cells. Polyadic synapses are highlighted with a dashed line. PPN = posterior pioneer neuron, PT = prototroch, PTC = prototroch cell. Scale bar: **(A–D)** 10 μm; **(E–H)** 100 nm.

In the stages investigated, the prototroch consists of 18 multiciliated cells (Figures 2A;B, Supplement 3A). Fourteen of these ciliated cells line up in one main transversal row, while two additional pairs of ciliated cells lie anteriorly to this row (Supplements 3A,B). In the 22 hpf stage, these two additional cell pairs are located anterior to cells 4 and 5/6, whereas in the 28 hpf stage, they are located anterior to cells 3/4 and 5. They probably correspond to the pairs of lateral ciliated cells described in other species of the annelid subgroup of Spionidae (Hannerz and Dag, 1956). The prototroch cells have a voluminous cell body and display long, strongly pigmented, and tapering basal processes (Figures 2A–D; Supplements 3C–E). These processes are reaching the nerve ring and may pass several other prototroch cells before they end (Supplement 3C–E). Synapses to the prototroch cells are located mainly on the basal processes.

### The PPN synapses to prototroch cells and extends further toward the apical plexus

The PPN is located at the posterior tip and consists of a large, elongated soma that sends two axons anteriorly and a dendrite posteriorly (Figures 1E,F, 2A–D). We could trace axons all the way up to the prospective head region. It became clear that the PPN axons extend further anterior than the prototroch already at 22 hpf. After passing the prototroch, the axons travel further anterior and end less than 5 μm away from the apical plexus. In 28 hpf, the distance to the plexus is even shorter, though this may be due to the extension of the plexus than by the ongoing growth of the axon. The axonal endings are evidently dilated in both stages, as is typical for axonal growth cones (Supplements 4A,B). We could, however, not find any filopodia extending from the axon endings. In the area where the axons pass the prototroch, we found synapses to basal processes of several prototroch cells in both stages (22 and 28 hpf; Figures 2C,D). High-resolution imaging of the synapses revealed that they contain predominantly small clear vesicles and a few larger dense core vesicles (Figures 2E–H; Supplement 6). In several cases, we noticed two adjacent postsynaptic cells on the opposite side of the presynaptic density resembling diadic/polyadic synapse morphology (Figures 2G,H; Supplement 6).

### The PNNs make contact with prototroch cells and join the PPN neural path toward the apical plexus

**Figure 3:**
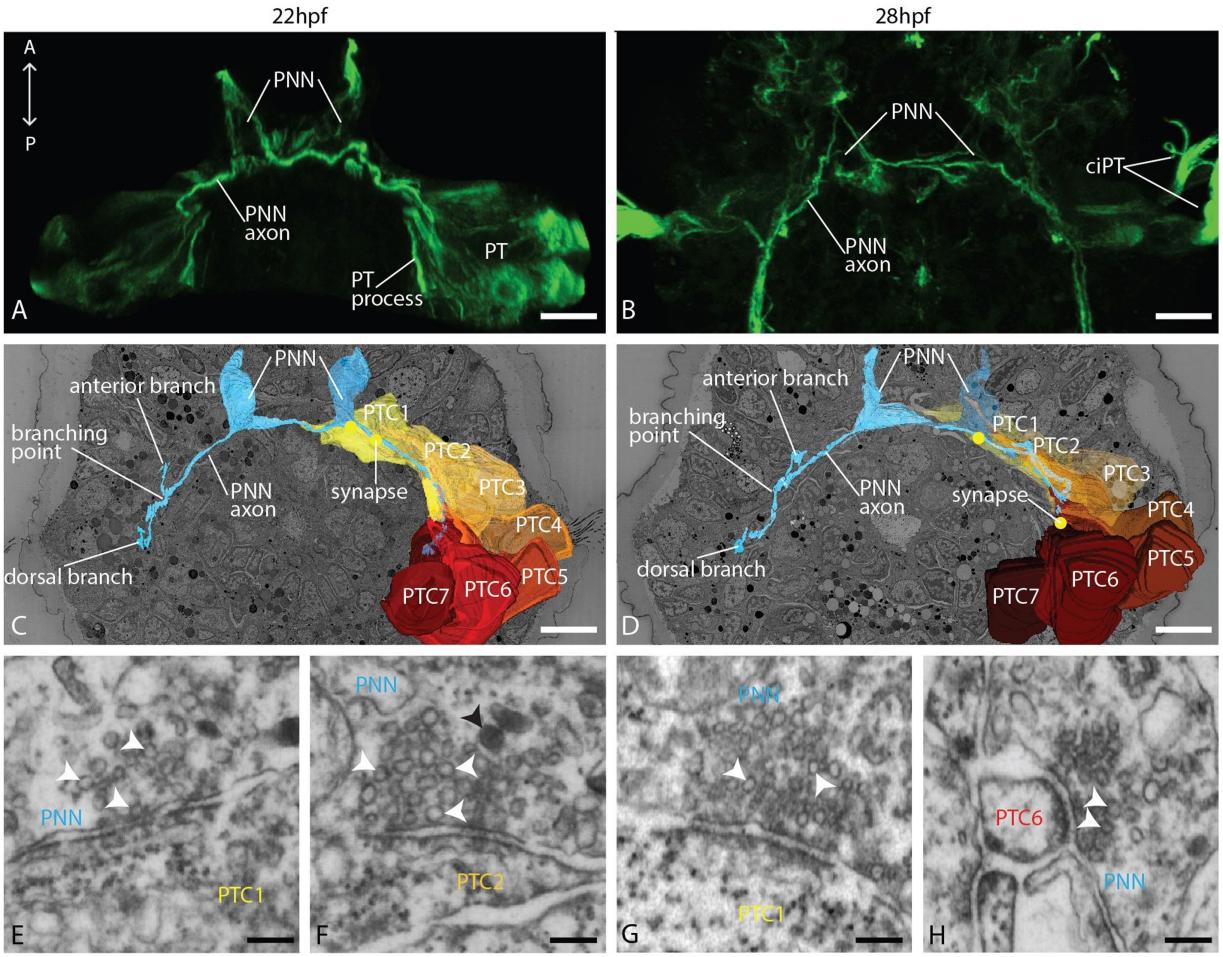
Organization of the PNN and the prototroch at 22 and 28 hpf. **(A,B)** CLSM, dorsal view of acetylated α-tubulin immunolabeled larvae showing the PNN and the prototroch; **(C,D)** dorsal view of 3D reconstructions of PNN, prototroch cells 1–7 (yellow to red) and selected synapses (yellow dots); and **(E–H)** detail of selected synapses between PNN and prototroch cells. PNN = prototroch nerve forming neuron, PT = prototroch, PTC = prototroch cell. Scale bar: **(A–D)** 10 μm; **(E–H)** 100 nm.

On the ventral body side, but slightly dorsal to the ventral prototroch cells, we identified left and right prototroch nerve-forming neurons (PNNs) described by Kumar et al. (2020) as a pair of triangular-shaped cells sending ciliated dendrites anteroventrally to the ventral body surface, and axons take the courses of the prototroch (Figures 3A–D). Their position and morphology correspond well in the EM and light microscopic data sets (Figures 3A–D). The few cilia extending from the dendrites taper to thin processes that run underneath the cuticle, as is common for chemo-sensitive neurons (Supplements 4D–F). While the extension of the PNN axons could not be revealed by light microscopy, we could fully trace them in the EM data set. Each of the PNNs sends two axons, one to the left and one to the right, underneath the prototroch cells (Figures 3C,D). Bending dorsally, they take the courses of the prototroch to the level of the crossing axon of the PPN. Here branch the axons of the left cell. One branch continues dorsally, while a second one runs anteriorly toward the apical plexus, joining the course of the PPN axon (Figures 3C,D; Supplement 3B). Similar and in close proximity to the tip of the PPN axons, the anterior branches of the left PNN axons end with a knob-like dilation resembling an axonal growth cone (Supplements 4A,B), while the dorsal branches are tapering without any dilation. Numerous synapses to the basal processes of prototroch cells are formed on the ventral part and in the dorsal branch of the axon (Figures 3E–H; Supplement 7). In contrast, the right PNN axons, which, according to Kumar et al. (2020), develop later than those of the left PNN, are not branching and extend only a little beyond the crossing with the PPN axon. In addition, the PPN axon forms synapses with the PNN axon (Supplement 4C) at the level where these axons are crossing.

### The AN1 receives synaptic input and synapses to the apical plexus and prototroch cells

**Figure 4:**
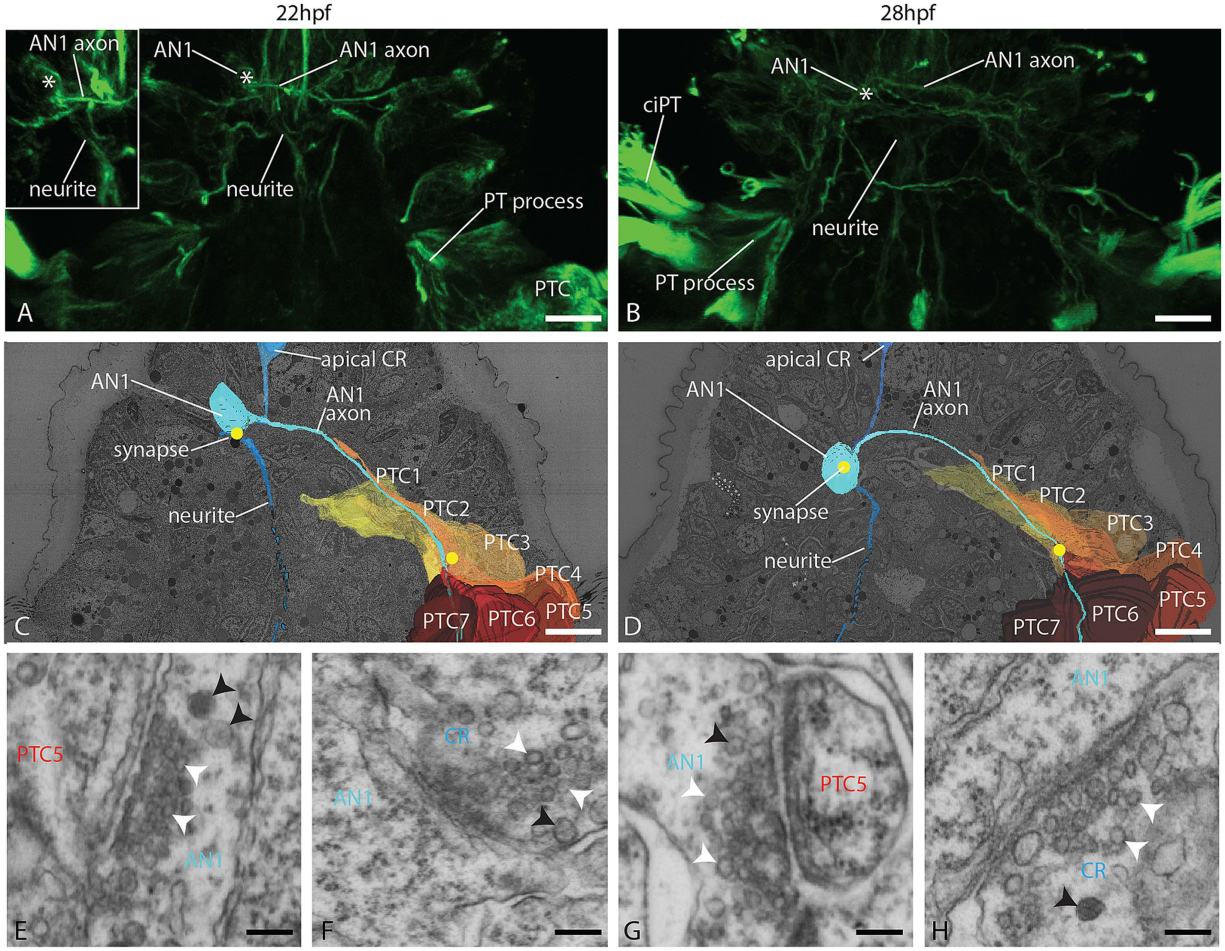
Organization of the AN1 and the prototroch at 22 and 28 hpf. **(A,B)** CLSM dorsal view of acetylated α-tubulin immunolabeled larvae, showing the AN1 and the prototroch. **(C,D)** A dorsal view of 3D reconstructions of AN1, prototroch cells 1–7 (yellow to red) and selected synapses (yellow dots); **(E–H)** detail of selected synapses between AN1 and prototroch cells; **(E,G)** and between AN1 and collar receptors **(F,H)**. AN1 = anterior neuron 1, CR = collar receptor, PT = prototroch, PTC = prototroch cell. Scale bar: **(A–D)** 10 μm; **(E–H)** 100 nm.

One cell layer dorsal and left to the apical tuft cell, we found a neuron without surface contact, which we term as a ganglion cell with a round cell body that does not contact the epidermal surface (Figures 1E,F). It sends a single axon through the apical plexus contralaterally to the right body side and extends further posterior. No other cell at this location has a similar morphology. By its position and projection, it corresponds to the AN1 described by Kumar et al. (2020) and the light microscopic data obtained (Figures 4A,B). In the EM data set, we could trace the axon far posterior. It meets the anterior end of the PPN and travels along this axon at least halfway down the trunk region (Figures 1E,F) or even further since we could not trace it until its end. In both stages, the AN1 axon forms several clear synapses to basal processes of prototroch cells (mainly to lateral prototroch cells; Figures 4C–E,G; Supplement 8). While passing the apical plexus, in the 24 hpf stage, it forms clear synapses to and from other neurites (Supplements 5A-C). The soma of the AN1 receives post-synaptic input from an axon belonging to one of the apical collar receptors described further below (Figures 4C,D,F,H; Supplement 8). We did not find any synapses between the AN1 cell and the apical tuft cell.

### Molecular and structural features point toward a multimodal function of the PPN

**Figure 5:**
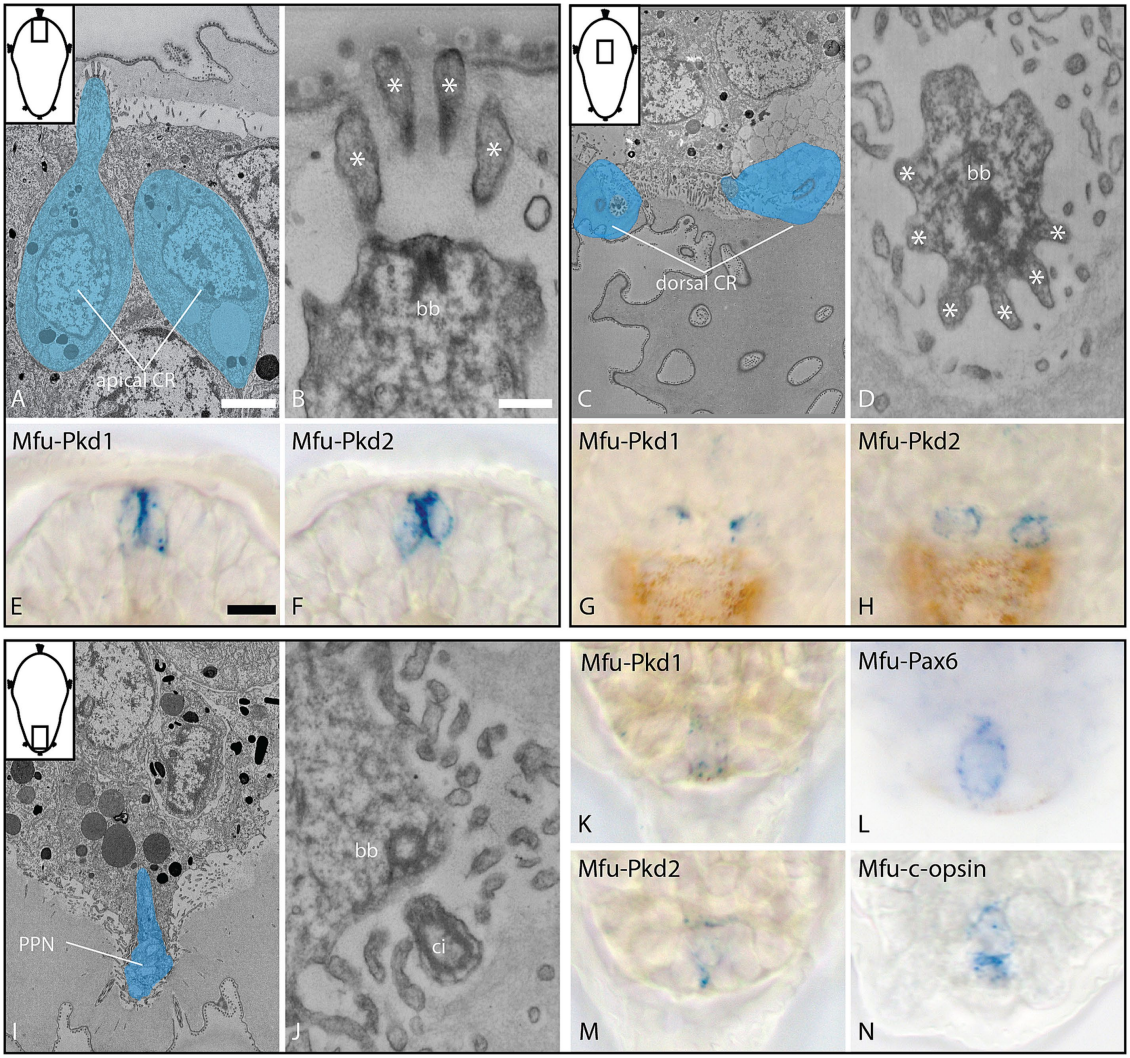
Ultrastructure and gene expression of collar receptor (CR) cells **(A–H)** and the posterior cell **(I–N)** at 28 hpf, dorsal view. **(A–D)** Overview and detail of apical CR cells (longitudinal section), expressing *Mfu-Pkd1* and *2*; **(E–H)** overview and detail of dorsal CR cells (cross-section, 3D outline), expressing *Mfu-Pkd1* and *2*; and **(I–N)** overview and detail of the posterior cell (longitudinal section), expressing *Mfu-Pkd1* and *2*, as well as *Mfu-Pax6* and *Mfu-c-opsin*. bb = basal body, ci = cilia, asterisks = collar microvilli. Scale bar: **(A,C,I)** 2 μm; **(B,D,J)** 200 nm; **(E–H,K–N)** 10 μm.

To further characterize the sensory nature of the PPN, we analyzed and compared the collar-like dendrite in the PPN, as well as in apical and dorsal collar receptor cells, using a combination of 3DEM and *in situ* hybridization. The dendrites of the apical and the dorsal cell pair each have a single cilium surrounded by a cuticle-penetrating, symmetric collar of 10 thick microvilli with a dense region on the inside, representing collar receptors (Figures 5A–D). The dendrite of the PPN, however, has four cilia surrounded by no or very thin microvilli, of which none reach the surface, providing no clear evidence (neither in longitudinal nor in cross-sections) for collar receptor-like organization (Figures 5I,J). At the base of the cilia is a symmetric anker of actin filaments located (resembling collar-like structures), underneath which basal bodies are detected. *In situ* hybridization experiments revealed clear expression of the mechanosensory markers *polycystin* (*PKD*) *1* and *2* in the apical and dorsal collar receptor cells, while the PPN showed clear expression of *PKD 1* and *2*, as well as the visual pigment *c-opsin* and the transcription factor *Pax6* (Figures 5E–H,K; Supplement 9).

## Discussion

With this study, we aim to better understand the role early neurons play in the development of the larval and the adult nervous systems in annelids with biphasic life cycles. For this purpose, we investigated the very early onset of neural development in the ciliated larvae of *Malacoceros fuliginosus* and looked for neuronal targets of the first neurons. These early neurons that send out long neurites to form the major routes of the later adult nervous system were identified as the main pioneer neurons in a previous study (Kumar et al., 2020). Neural development in the larvae of this species starts on the anterior and posterior pole of the organism, as it is documented also for several other annelids with a biphasic lifecycle (Fischer et al., 2010; McDougall et al., 2006; Starunov et al., 2017; Voronezhskaya et al., 2003). While in all these species numerous neurons emerge in the anterior region in a short time interval and later give rise to prominent structures like the apical nerve plexus and a broad set of peripheral sensory neurons, a single neuron precedes the formation of additional neurons in the hind end for a longer period. This first neuron in the posterior regions sends out two axons, which grow anteriorly on the ventral side of the trunk.

### The early neurons have both a pioneering and a signaling function

Though neurotransmitter expression has been documented in several species (Fischer et al., 2010; McDougall et al., 2006; Starunov et al., 2017; Voronezhskaya et al., 2003), light microscopic studies could not uncover whether the posterior pioneer neuron, in addition to its path-finding role, also establishes synaptic contacts to other neurons. First evidence was provided by electron microscopic studies in 72-h-old larvae of *Platynereis dumerilii*, wherein the nervous system is already quite complex (Bezares-Calderón et al., 2018; Randel et al., 2014; Verasztó et al., 2017) with a prototroch that receives synaptic input by many motoneurons. The posterior neuron (pygPB^unp^), however, is special. It is in this developmental stage one of only three sensory neurons, which directly synapse to ciliated cells of the prototroch and, accordingly, are categorized as sensory-motoneurons. While a sensory function is well documented for the other two sensory-motor neurons (the eyespot photoreceptor cells; Jékely et al., 2008; Randel et al., 2014), these do not pioneer major developing nerves.

We see in *M. fuliginosus* that the posterior pioneer neuron (PPN), which has a similar course as the pygPB^unp^ in *P. dumerilii* (Kumar et al., 2020), also makes synaptic contact with prototroch cells. And by focusing on very early stages, we see synapses already 22 h after fertilization, shortly after the extending axons reach the level of the prototroch. This connectivity pattern is, however, not a peculiarity of the PPN in this stage. On the contrary, it is true for all the early pioneer neurons we identified. The anterior pioneer neuron (AN1), which connects the anterior NS to the trunk ventral NS, and the two pioneer neurons forming the nerve underneath the prototroch (PNNs) also make synaptic contact with prototroch cells.

Hence, besides their function in establishing major nerves, all the examined pioneer neurons in *M. fuliginosus* are (cilio)motor neurons that likely participate in regulating the beating pattern of the prototroch—the primary larval locomotory organ. To definitively confirm that these early neurons indeed serve as pioneers and to assess their influence on follower neurons, cell ablation studies at different larval stages will be essential.

### The prototroch receives very early sensory input from several modalities

Due to the hard-wired input by the pioneers, the swimming activity of the larva may respond quickly already very early in development. These neurons probably sense a broad spectrum of environmental stimuli. The extension of dendritic cilia underneath the cuticle points toward a chemo-sensory function of the PNNs. The AN1 likely transmits mechanical stimuli to the prototroch due to the synaptic input from a neuron that expresses the mechanosensory markers *polycystins 1* and *2* and exhibits a typical collar receptor morphology. Even though the dendritic cilia of the PPN deviate from the typical collar receptor morphology, we assume that it likewise responds to mechanical stimuli, since it also expresses *polycystin 1* and *2* that we found expressed in regular collar receptors. Interestingly, the morphology of the PPN resembles the type-1 sensory cells that accompany the collar receptors in annelid lateral organs (Purschke and Hausen, 2007). The co-expression of the visual pigment *c-opsin* and the transcription factor *Pax6* further suggests light sensitivity and, hence, a multimodular sensory function. While nonvisual opsins are not uncommon in protostomes, the presence of a ciliary opsin in the PPN is indeed intriguing. Two ciliary opsin paralogs are known from annelids. The *Platynereis c-opsin* has been studied intensely, and functional characterization assigned it with a UV sensing function (Arendt et al., 2004; Tsukamoto et al., 2017) and an involvement in barotaxis (Bezares Calderón et al., 2023). In contrast, the spectral characteristics of *Platynereis c-opsin2* suggest a shadow reflex function (Ayers et al., 2018), but it remained elusive in which cells it is expressed. *Mfu-c-opsin* is the ortholog to *Platynereis c-opsin2* (Döring et al., 2020), and we see this type of visual pigment in *M. fuliginosus* expressed at the very hind end of the larva in the PPN.

The PPN and AN1 are likely not only involved in direct signaling. The multiple synaptic contacts the PPN makes with the axon of the PNN and the AN1 makes with other anterior neurites suggest that these cells also have a modular function.

### The pioneer neurons may target additional cells later in development

Notably, the prototroch is probably not the final target of the pioneer neurons, since all of them synapse without forming proper terminal endings while passing (en passage) to the ciliated prototroch cells. The posterior pioneer neuron extends further anterior, and the anterior pioneer neuron runs further posterior. At least the left PNN is branching at its lateral-most position and sends one branch to the anterior body region. Whether the right cell, which develops more slowly than the left one (Kumar et al., 2020), does the same in later stages remains unclear. The growth cone-like swellings on the tips of the PPN and the left PNN axons suggest that they can extend even further in search of their final destination, or they lie dormant until potential target neurons develop. This might be the case for the PPN. Though the axons show growth cone-like swellings, they barely extend more anteriorly in the 28-hourstage than in the 22-h stage. In the 72-h stage of *P. dumerilii,* the corresponding pygPB^unp^ synapses not only to head prototroch cells but also to different kinds of pigmented cells and to trunk sensory, inter-, and motoneurons (Jékely et al., 2024).

Remodeling of neural circuitry during development is a common phenomenon. The extent to which this can occur has recently been shown by developmental connectomic studies in *Caenorhabditis elegans* (Witvliet et al., 2021). While sensory and motor pathways change considerably, decision-making circuits remain fairly stable. In annelids, developmental change of circuitry has not been systematically studied, nor have many individual neurons been traced through metamorphosis. The few data existing are mainly from the first pair of eyes occurring in development, whose photoreceptor cells in 48-h larvae mediate phototactic behavior by direct signaling to prototroch cells (Jékely et al., 2008), whereas the same cells in later stages synapse also to motoneurons of the optic neuropil (Randel et al., 2015). Our data raises the question of whether the pioneer neurons described undergo similar changes in their connectivity later in development.

Even the organization of the synapses might change over time. In the early stages, we studied how the PPN forms diadic synapses to the prototroch cells. Di- and polyadic synapses are common in many invertebrate organisms (Brittin et al., 2018; Cardona et al., 2010; Meinertzhagen, 2017; Ryan et al., 2016). According to Liu et al. (2007), polyadic synapses represent a distinct mechanism for synaptic divergence and for synchronizing the activities of postsynaptic cells. In the 72-h-old stage of *P. dumerilii*, synaptic connectivity relies on, however, completely on monadic synapses. We cannot rule out that it is similar in older stages of *M. fuliginosus*, when navigation and possibly the regulation of the prototroch ciliary beating pattern become more complex.

### Did the pioneer neurons in the first place serve the adult or the larval stage?

In most indirectly developing annelids, the transition from the larval to the adult body organization, as in *M. fuliginosus*, is a gradual process. With respect to the nervous system, this is generally understood as the conservation of core elements and the loss of larval neural elements. Due to the course and connectivity of the investigated neurons in *M. fuliginosus*, it is, however, difficult to classify the cells as either being elements of the larval or of the prospective adult nervous system. The PPN and the AN1 target a purely larval organ, which is cast during metamorphosis, but they also lay out the route of the CNS, which survives metamorphosis (Kumar et al., 2020). Furthermore, the axons extend beyond the prototroch and may later connect to other targets. Seemingly, these cells are important for both the larval and later developmental stages.

How did this evolve? Are these cells descendants of neurons with a primary function in controlling the locomotion of swimming larvae, and did the adult CNS evolve along the routes these cells establish in the embryo? This would be in accordance with the larva-first hypothesis (Marlow et al., 2014; Martín-Zamora et al., 2023; Nielsen, 2013; Peterson et al., 1997) of animal life-cycle evolution.

However, the data do not rule out the alternative hypothesis, i.e., that larval forms arose by the intercalation of larval features in the development of an ancestral direct-developing organism (Page, 2009; Raff, 2008; Wang et al., 2020). The first neurons could have had a primary function in outlining the adult nervous system and primarily made contact with their adult targets. On their way, they formed en passage synapses to the ciliated cells. And due to positive selection for early control of larval swimming, the prototroch cells became the first targets of the neurons.

Comparative data from other organisms are needed for giving support to one or the other hypothesis on the evolution of life cycles.

### What is known from other taxa?

In several annelids, a neuron with similar bi-axonal morphology as the posterior pioneer neuron in *M. fuliginosus* has been found (Fofanova et al., 2021; McDougall et al., 2006; Starunov et al., 2017; Voronezhskaya and Elekes, 2003; Voronezhskaya et al., 2003), though it is apparently absent in some species like *Capitella teleta* (Meyer et al., 2015) and respective data are missing from several early derived annelid groups. Based on the ultrastructural and molecular data from *P. dumerilii* and *M. fuliginosus*, two species of the large annelid subgroups of Errantia and Sedentaria (Struck et al., 2011; Weigert and Bleidorn, 2016), we infer that this neuron had a mechanosensitive function, pioneered the ventral nerve cord, made synaptic contact to the prototroch, and extended further toward the brain already in the last common ancestor of the majority of the annelids. It will be interesting to figure out whether the respective “adult” neuronal targets in later developmental stages also correspond to each other and how this compares to the situation in direct developing annelids. Dinophiliids provide an example that the course of early nervous system development can be well preserved in pedomorphic indirect developers (Fofanova et al., 2021).

The anterior–posterior outgrowth of neurites from the apical tip toward the later ventral nerve cord is likewise well documented by immunohistochemical studies in many annelids (McDougall et al., 2006; Starunov et al., 2017; Voronezhskaya et al., 2003). A more detailed comparison to the situation in *M. fuliginosus* is, however, difficult in many cases. Common neurotransmitter staining reveals only a subset of the existing neurons, and surface-connecting sensory and underlying ganglion neurons of the apical plexus develop in close vicinity. In contrast to descriptions of many other annelids, the first neurite growing posteriorly originates in *M. fuliginosus* not from a surface-contacting sensory cell but from a ganglion cell, which lies directly underneath and receives axonal input from a flask-shaped sensory cell. It may need data with pan-neural markers or volume EM in the early stages of other species to figure out whether neurogenesis in the apical region of *M. fuliginosus* deviates from the common pattern or not. A comparison of the synaptic connectivity of the apical pioneering neurons would be desirable, but no data other than those from *M. fuliginosus* are available yet.

The nerve ring underneath the prototroch in *M. fuliginosus* is not prefigured by axons extending from the apical or posterior pole but by the two PNNs that lie adjacent to the ventromedial prototroch cells (Kumar et al., 2020). So far, this has not been observed in other annelids. Which does not necessarily mean that it is different due to the stages investigated in the studies or the reliance on few neurotransmitter staining, which only labels subsets of the neurons existing. Even in detailed studies on species like *P. dumerilii* (Starunov et al., 2017), it remains unclear from which cells the first neurites underneath the prototroch originate. Yet, the cells whose axons first run around the whole prototroch are catecholaminergic neurons, which have a similar position as the PNNs in *M. fuliginosus*.

A substantial record of immunohistochemical data shows many similarities in the nervous system development between annelids and other lophotrochozoan/spiralian larvae. The main routes of the nervous system are likewise formed by neurites of only a few neurons. The early appearance of anterior and posterior neurons pioneering the main longitudinal routes of the later nervous system has been found in larvae of several groups of mollusks (Battonyai et al., 2018; Croll and Voronezhskaya, 1996; Pavlicek et al., 2018; Redl et al., 2014; Voronezhskaya and Elekes, 2003; Yurchenko et al., 2018, 2019) and also nemerteans (Hiebert and Maslakova, 2015; Von Döhren, 2024) and flatworms (Rawlinson, 2010).

ConclusionComparative data on the synaptic connectivity of the first neurons in larvae will be valuable for a better characterization of the nervous system in groups with ciliated larvae. Such data will provide the basis for investigations into the neurobiological and behavioral capabilities of early larvae. It will clarify which components serve the larval stage, the adult stage, or both, and how the larval and the adult nervous systems can be considered independently developing entities. Additional data from direct developers would be greatly interested in this context. Furthermore, we anticipate that early circuitry data, combined with emerging molecular insights into early development, will enhance understanding of animal life cycles and larval evolution. Conservation of the adult targets of the first neurons will support the adult-first hypothesis (Page, 2009; Raff, 2008; Wang et al., 2020), and conserved patterns in the establishment of the larva-specific circuitry will support the larva-first hypothesis (Marlow et al., 2014; Martín-Zamora et al., 2023; Nielsen, 2013; Peterson et al., 1997). Heterochronic modifications in larval development, which may account for the (multiple) emergence of larvae with an expanded anterior domain (Gonzalez et al., 2017; Martín-Zamora et al., 2023; Strathmann, 2020), probably also contribute to the connectivity of early neurons.

## Data Availability

Publicly available datasets containing the PKD1 and PKD2 sequences were analyzed in this study. This data can be found on Genbank with accession numbers PQ818802 and PQ818803.
