# Supplementary material for: Neurons with larval synaptic targets pioneer the later nervous system in the annelid Malacoceros fuliginosus
Source: Front Neurosci. 2025 Jan 13;18:1439897. doi: 10.3389/fnins.2024.1439897 (PMC11770012; doi:10.3389/fnins.2024.1439897)
Supplement: Supplementary file 1 [file Image_1.pdf]

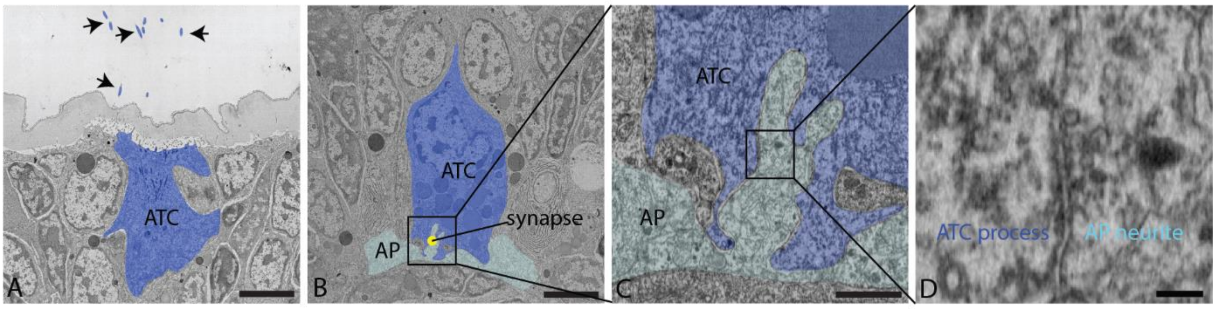

Supplement 1. Organization of the apical tuft cell of 22h larvae. **(A)** apical part of the apical tuft cell with cilia (blue, arrowheads); **(B-D)** overview and details of the basal part of the apical tuft cell, the apical plexus (light blue) and the synapse between them. AP = apical plexus, ATC = apical tuft cell. Scale bar: A,B, 5µm: C, 1µm; D, 100nm.

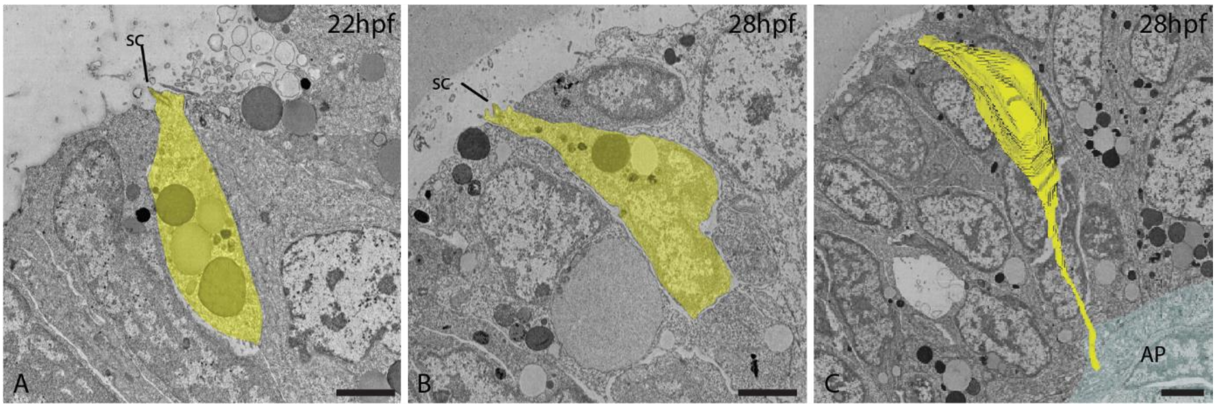

Supplement 2. Flask shaped sensory cells of the anterior epidermis of 22 and 28hpf larval stages. **(A,B)** epidermal sensory cell with sensory cilium (yellow); **(C)** 3D reconstruction of the epidermal sensory cell with neurite towards the apical plexus (light blue). AP = apical plexus, sc = sensory cilium. Scale bar: 2µm.

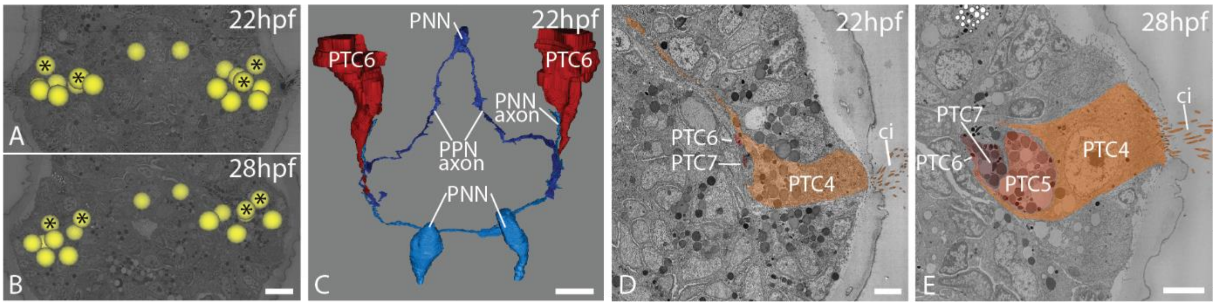

Supplement 3. Organization of the prototroch of 22 and 28hpf larval stages. **(A,B)** 3D reconstructions showing the position of the nuclei of the 18 multi-ciliated cells forming the prototroch with ciliated cells lying slightly anterior to the main row (arrows); **(C)** anterior view of the PPN (dark blue), PNN (light blue) and selected prototroch cells (PTC6, red); **(D,E)** prototroch cells with cilia and processes shown. ci = cilia, PNN = prototroch nerve forming neuron, PPN = posterior pioneer neuron, PTC = prototroch cell. Scale bar: A,B, 10µm; C-E, 5µm.

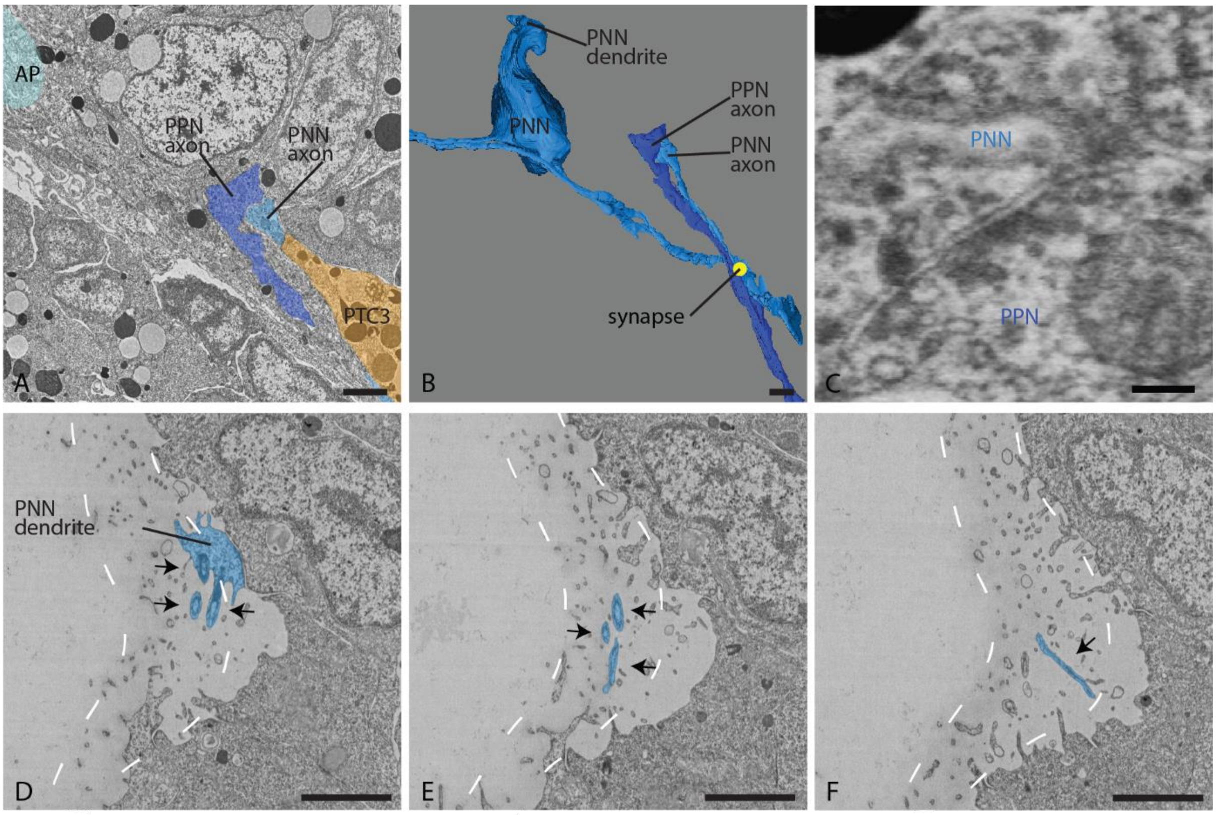

Supplement 4. Details of dendrites and axons of the PPN and PNN of the 28hpf larval stage. **(A)** nob-like dilations of the PPN and PNN axons; **(B)** 3D reconstruction showing the crossing of the PPN and the PNN axons, a synapse and the PNN dendrite; **(C)** detail of the synapse between the PPN and PNN axons; **(D-F)** detail of the PNN dendrite with cilia (arrows) tapering to thin processes which run underneath the cuticle (dashed white line). AP = apical plexus, PNN = prototroch nerve forming neuron, PPN = posterior pioneer neuron, PTC = prototroch cell. Scale bar: A,B,D-F) 2µm; C, 100nm.

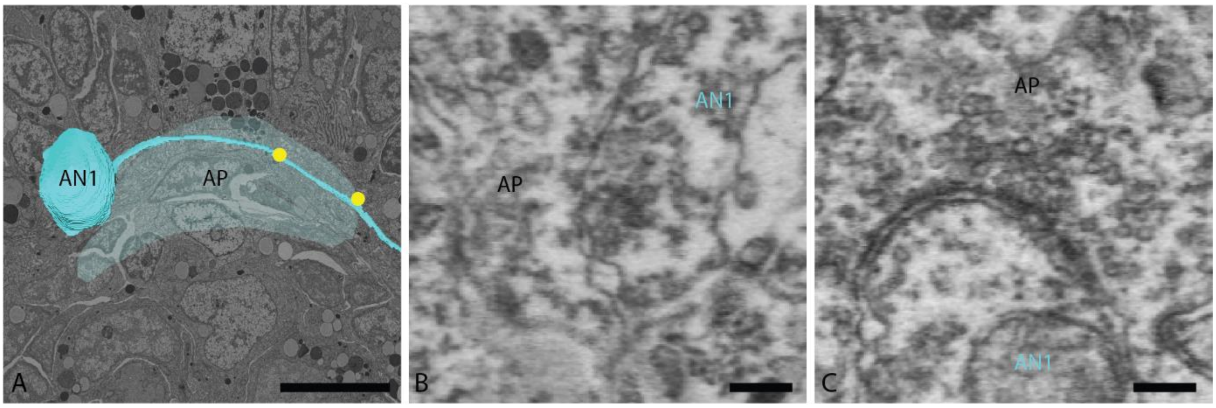

Supplement 5. Pre- and postsynaptic structures of the AN1 axon of a 28hpf larval stage. **(A)** 3D reconstruction showing an overview of the AN1, apical plexus and synapses (yellow dots); **(B,C)** detail of AN1 pre- and postsynaptic structures, respectively. AP = apical plexus. Scale bar: A, 10µm; B,C, 100nm.

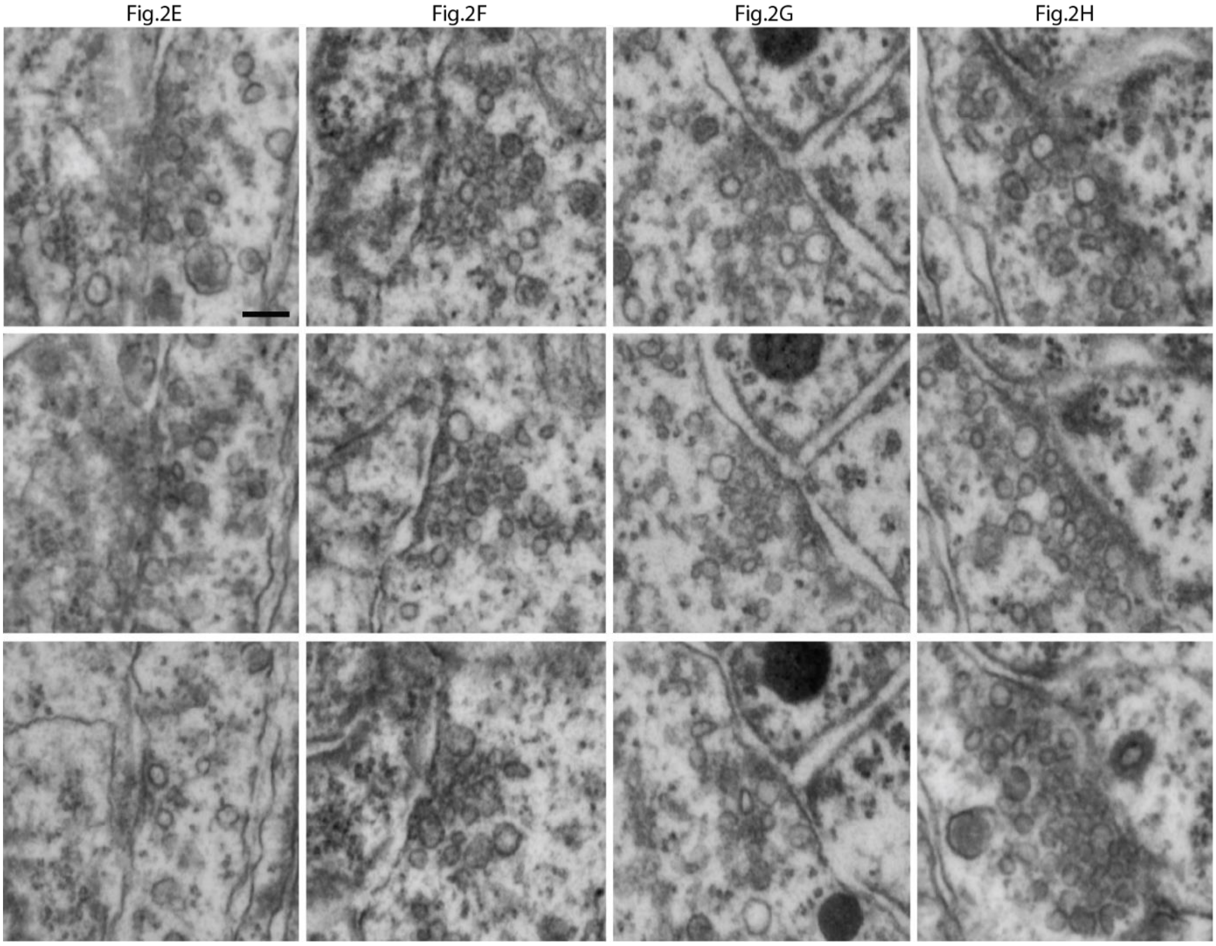

Supplement 6. Series of sections through synapses displayed in Figure 2E-H.

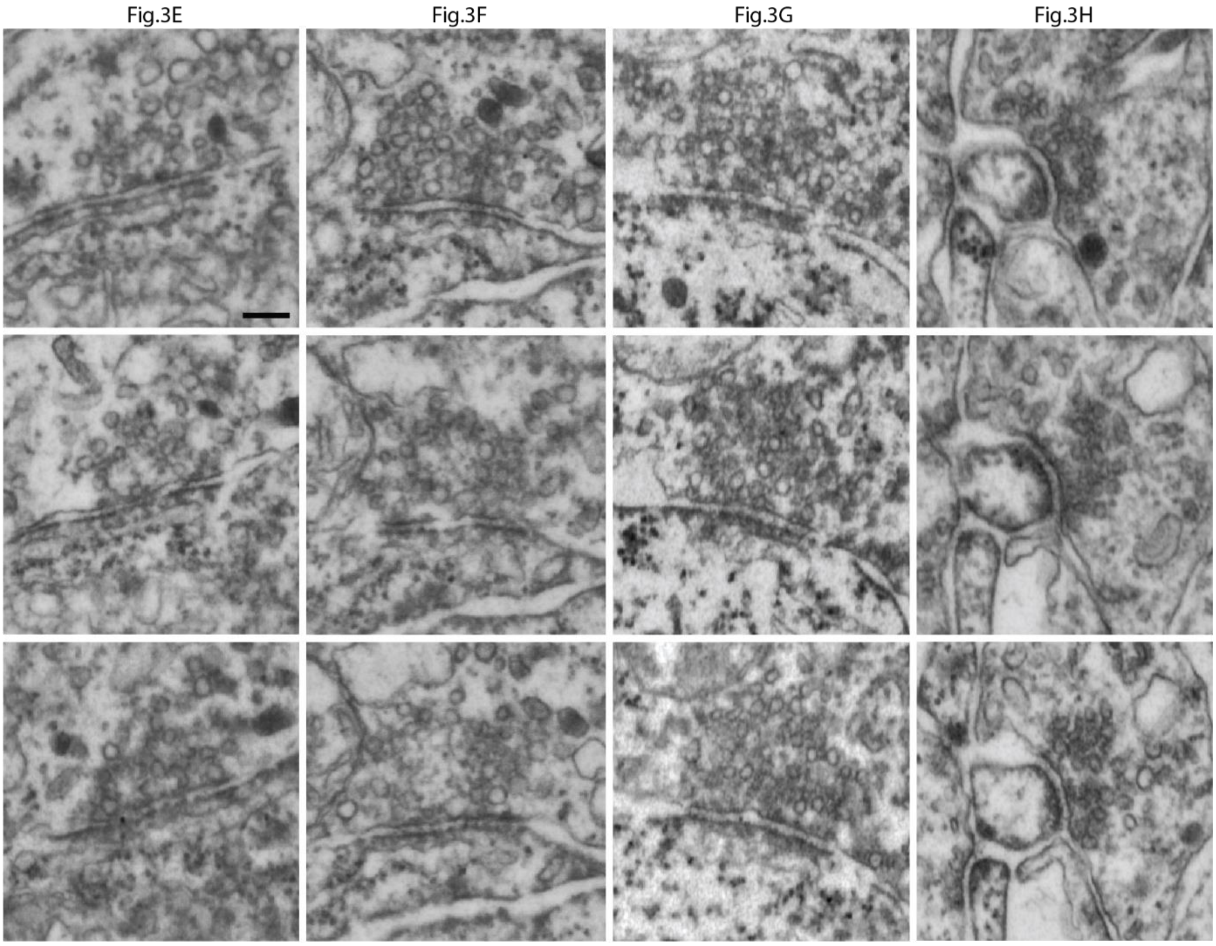

Supplement 7. Series of sections through synapses displayed in Figure 3E-H.

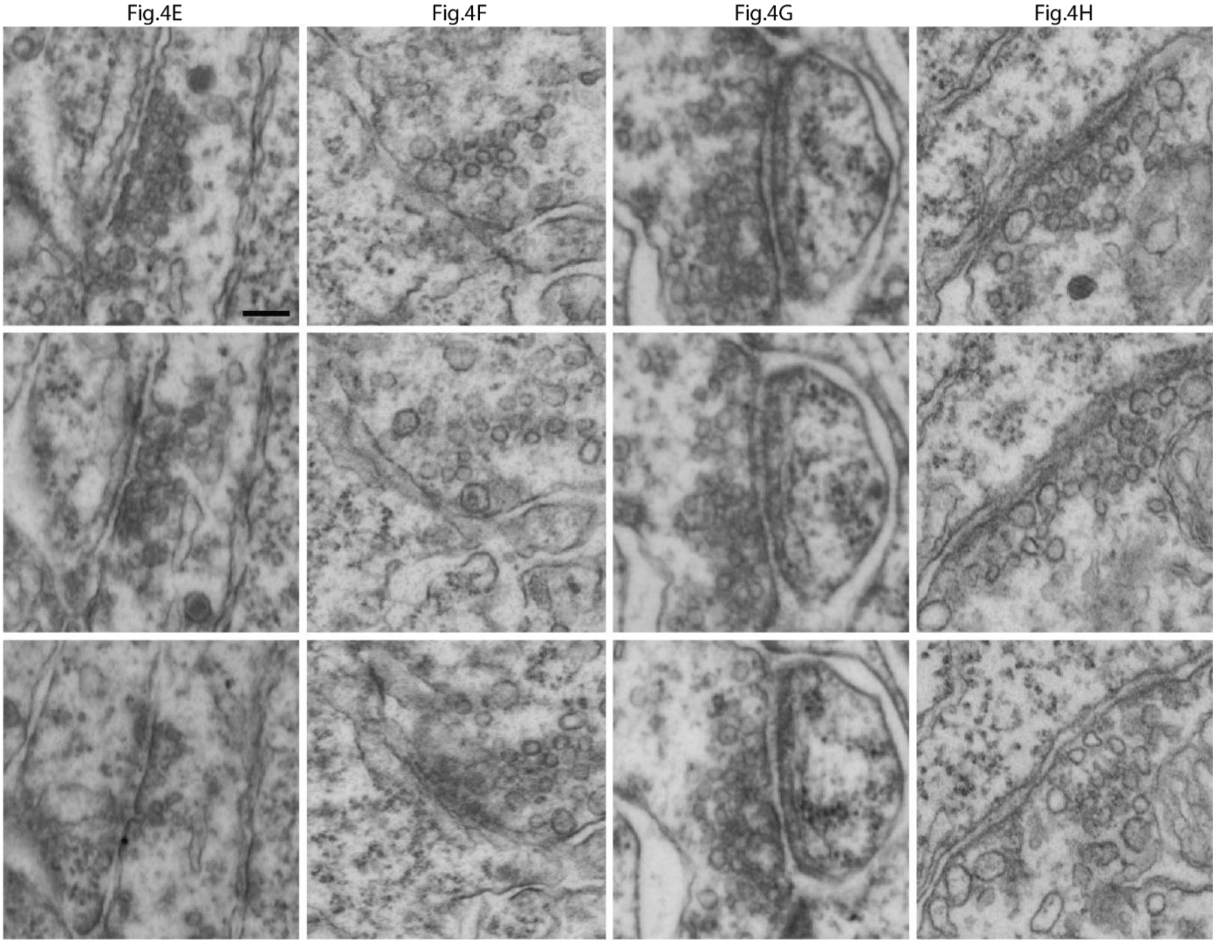

Supplement. 8. Series of sections through synapses displayed in Figure 4E-H.

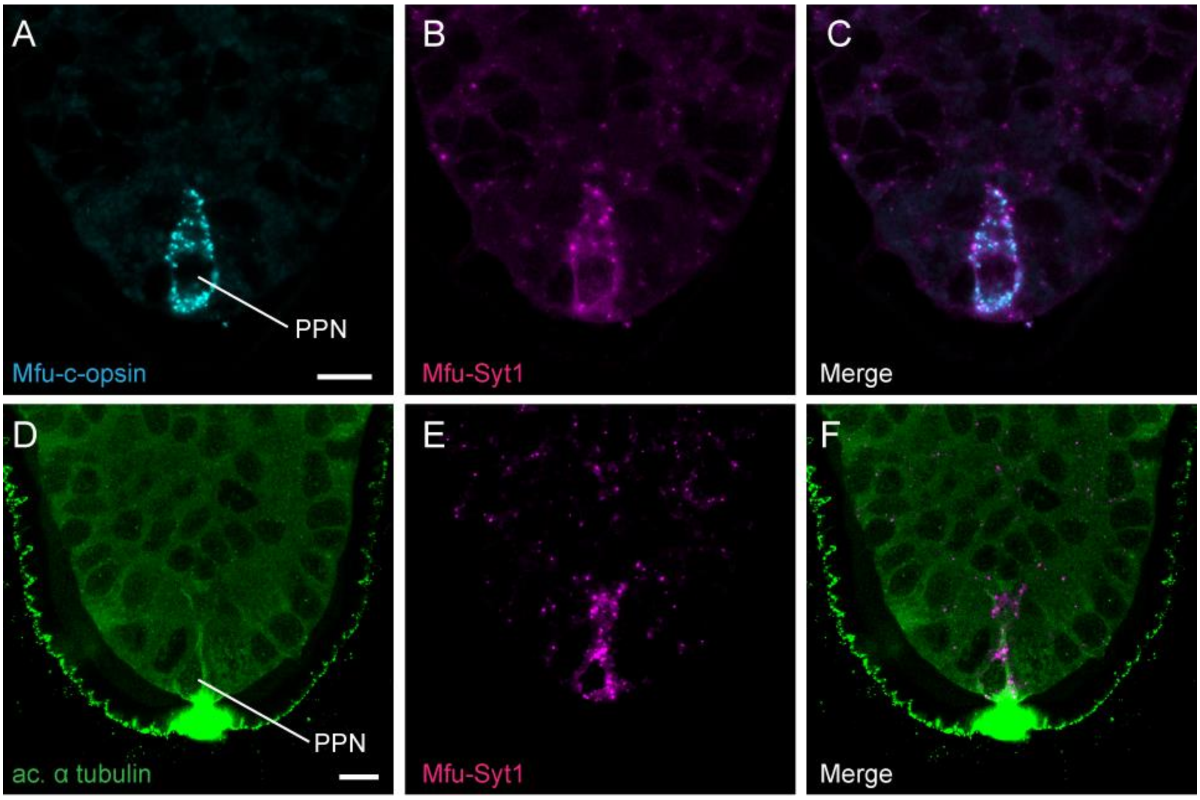

Supplement. 9. Co-expression of Mfu-c-opsin and Mfu-Syt1 in the PPN (A-C) and co-expression acetylated α-tubulin and Mfu-Syt1 in the PPN (D-F) in 14hpf larval stage. PPN = posterior pioneer neuron. Scale bar: 10μm.
